# Marked increase in measles vaccination coverage among young adults in Switzerland: a campaign or cohort effect?

**DOI:** 10.1007/s00038-018-1102-x

**Published:** 2018-04-19

**Authors:** Ekkehardt Altpeter, Monica N. Wymann, Jean-Luc Richard, Mirjam Mäusezahl-Feuz

**Affiliations:** Division of Communicable Diseases, Federal Office of Public Health, Berne, Switzerland

**Keywords:** Measles, Vaccination, Vaccination coverage, Epidemiology, Survey

## Abstract

**Objectives:**

To evaluate the impact of the Swiss measles elimination strategy—including a mass media campaign—on vaccination coverage and awareness among young adults aged 20–29 years.

**Methods:**

Comparison of the results of two cross-sectional population surveys in 2012 and 2015.

**Results:**

Documented vaccination coverage increased from 77 to 88% for two doses of measles vaccine. Major determinants of complete vaccination were survey year, birth cohort, sex and the absence of prior measles disease. If birth cohort and prior history of measles disease are included as factors in a multivariate model, the difference between 2012 and 2015 vanishes.

**Conclusions:**

The marked increase in complete measles vaccination coverage is due to a cohort effect, owing to the introduction of the second dose of vaccine in 1996. Most of the vaccinations were administered before the national strategy was implemented and vaccination catch-ups did not increase during the campaign in young adults. Nevertheless, this study provides evidence of an improvement in the awareness of measles and measles vaccination in young adults, which may result in an impact on measles vaccination coverage in the near future.

**Electronic supplementary material:**

The online version of this article (10.1007/s00038-018-1102-x) contains supplementary material, which is available to authorized users.

## Introduction

Measles is a highly transmissible infectious disease. Its complications are particularly frequent and severe in infants and adults. However, it is possible to prevent the disease effectively with vaccination. The Swiss Federal Office of Public Health (FOPH) has recommended the vaccination of 12-month-old children against measles since 1976, and has recommended the combined vaccine against measles, rubella and mumps since 1985. In 1996, a second dose was added to the vaccination schedule for 4–7-year-old children; in 2001, the recommended age for this second dose was reduced to 15–24 months.

Despite a relatively high vaccination coverage of 93% with two doses during the years 2014–2016 in the 16-year-olds, measles is still an endemic disease in Switzerland. The annual baseline incidence is low at 3–10 cases per million people. However, a large outbreak between 2007 and 2009 led to over 4000 cases (Richard and Masserey Spicher 2009). On this occasion, Switzerland also exported measles, causing outbreaks in various European countries and the United States. In response to this national outbreak and to achieve the WHO’s goal of eliminating measles in the European region by 2015, the FOPH together with the cantons (states) and other public health partners launched a national strategy for 2011–2015 to eliminate measles (Bundesamt für Gesundheit 2013, 2016a). One of the major objectives of this strategy was to raise public awareness of measles and vaccination against this disease to sustainably increase two-dose vaccination coverage to at least 95% in children and adolescents, including through catch-up vaccinations for individuals born after 1963. The strategy consisted of six areas of intervention, including facilitating and encouraging catch-up vaccinations in the over 2-year-olds to fill vaccination gaps, as well as communication and promotion to raise public awareness of measles and vaccination against this disease.

Although vaccination coverage for measles has continually increased in children of all age groups since 2000, the national target of 95% of people vaccinated with two doses has not yet been reached. According to the last cantonal surveys of 2-, 8- and 16-year-olds during the period 2014–2016 (Swiss National Vaccination Coverage Survey—SNVCS), national coverage for a single dose was 94–96% depending on age group, but only 87–93% for two doses (Bundesamt für Gesundheit 2016b). However, a few cantons did reach 95% for two doses. Vaccination coverage of adults is not routinely monitored in Switzerland and is unknown.

The aim of this study was to assess the effect of the measles campaign in regard to improved vaccination coverage in young adults by catch-up vaccinations and increased awareness. With two consecutive surveys, we assessed the vaccination coverage as well as awareness of measles in 20–29-year-olds in 2012 before the campaign (baseline) and in 2015 afterwards (endline).

## Methods

### Study design

We designed two identical nationally representative cross-sectional telephone surveys before and after the campaign in 2012 and 2015, respectively, conducted by the same commercial telephone survey company, using identical sampling methods and assessment tools for both baseline and endline survey. The same professional telephone survey company conducted recruitment and data collection for both surveys. The interview included the same questions regarding: participants’ self-reported measles vaccination status, reasons for receiving measles catch-up vaccination, willingness to be vaccinated if vaccinations were missing and measles disease history, as well as knowledge of the disease and the elimination campaign (for the wording of the questions see Table 3). All participants were asked to send a copy of their vaccination card to the Federal Office of Public Health. The average interview time was about 7 min. The questionnaire was available in German, French and Italian. The sampling procedure and questionnaire were piloted with 38 completed interviews. The baseline survey took place between April 23 and July 22, 2012 and the second survey took place between November 9 and December 18, 2015.

The two surveys were conducted under the Swiss Epidemics Act of December 18, 1970, thus ethical committee approval was not required.

### Participants, recruitment and sample size

Inclusion criteria for participation in the study were: age 20–29 years, address within Switzerland recorded in a commercial directory, and ability to answer questions in German, French or Italian. The survey company used a commercial household directory, which covers about 95% of all Swiss private households. Stratified random sampling based on two (baseline) or three (endline) language regions and municipality size was applied. Each selected household received an invitation letter from the FOPH to announce the study and its background. A total of 8645 invitation letters were sent. For households without a registered telephone number, the letter requested for it. A free telephone hotline for participants who wished to know more about the study was in place during both study periods.

The telephone contact started with a screening for willingness to participate and to assess eligibility by age. If several persons within a household fulfilled the age criteria, one was selected at random. At the end of the telephone interview, respondents were asked to send their vaccination card or a copy of it to the FOPH. The originals were returned after data collection. Those consenting to send in their vaccination card received an instruction letter with a stamped and addressed envelope and an incentive of CHF 10. If the FOPH did not receive the vaccination card within 3 weeks, a reminder was sent. To get the vaccination cards, a total of 2717 letters were sent followed by 1373 reminders.

We anticipated a ratio of one vaccination card received per two completed telephone interviews. The number of attempted interviews was calculated on this basis. The target sample size was 350 interviews with matching vaccination cards per language region. We aimed at a total sample size for interviews with matching vaccination cards of 700 in the baseline with two language regions and 1050 in the endline with three language regions.

### Statistical analyses

We calculated sampling weights according to the same protocol for both surveys: The sampling frame was a database that covers 95% of all households in Switzerland. Weights accounted for each language region and were then calibrated to the population total considering sex and canton (Lumley 2004, 2014a).

**Fig. 1:**
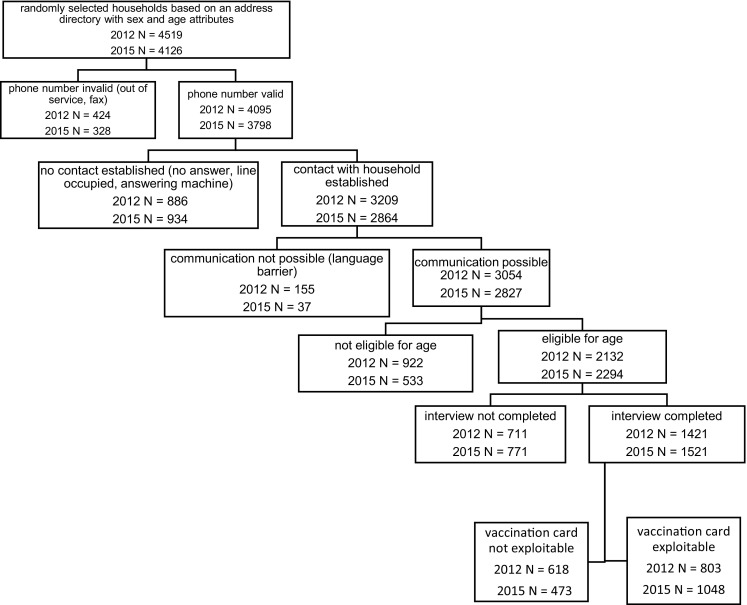
Data flow chart, measles surveys 2012 and 2015, Switzerland

We combined the data of the two surveys and defined an indicator variable identifying the 2012 survey as zero and the 2015 survey as one. We will call this variable ‘survey’ in the following. Since we did not sample data from the Italian-speaking part of Switzerland in the 2012 survey, we discarded these data from the 2015 survey for this analysis. Only datasets with a vaccination card were considered for the analysis, resulting in an overall total *N* of 1851 (Fig. 1).

The primary endpoint was defined as documented vaccination with at least two doses of measles vaccine. We defined two cohorts based on the median birth year of the complete study population: one born in 1990 or later formed the reference group, while the other cohort included respondents born before 1990. In a sensitivity analysis, we included annual birth cohort as a factor into the analyses. We recoded nationality for 2015 to the standard of 2012, since in 2012 we had two groups (Swiss and foreign nationality) and in 2015 three groups (Swiss, foreign and both). We defined people with both nationalities as Swiss.

We analysed the data excluding and including the sampling weights. For unweighted analysis, we used standard tabulations and standard inference. The level of significance was set to alpha < 0.05. We calculated 95% confidence intervals (CI) where applicable. For the weighted analysis, we described the data by means of tabulation and calculated proportions (Thomas Lumley 2004, 2014a).

We evaluated the primary endpoint in a univariate analysis and then included all variables with a significant effect in a multivariate logistic regression model considering the survey weights and calculating odds ratios (OR) and their 95% CI. This analysis assumes ‘missing completely at random’ (MCAR).

All logistic regression models (weighted as well as unweighted) were estimated with and without multiple imputations assuming a joint model (Quartagno and Carpenter 2016). The model included all categorical variables as well as age and weight as continuous variables. We estimated five imputed datasets and combined the results by means of Rubin’s rule (Lumley 2014b).

We tabulated the documented vaccine coverage by cohort and the variable ‘survey’. We determined the vaccination coverage at age 16-years by cohort and compared these estimates with coverage of the SNVCS for that age. We further derive the catch-up vaccination by subtracting the overall cohort-specific vaccine coverage at the age of 16 years from the overall cohort-specific vaccine coverage at the time of the interview. We restricted our analysis to the birth cohorts 1986–1992 which overlap between the two surveys and compared the vaccination coverage for at least two doses at the time of interview between the survey 2012 and 2015. We included annual birth cohort as a factor in a logistic regression model.

To evaluate the effect of absent vaccination cards, we tabulated its absence and presence, respectively, with respect to the following variables: ‘survey’, sex, age, language region, anamnestic vaccination status, prior measles history and estimated a linear unweighted logistic regression model.

We performed all analyses using R for Windows 3.1.3, respectively, 3.3.1 under Windows 7, made available by the R Core Team (2015).

## Results

### Response rate and basic characteristics of participants

The response rate for completed interviews was 50% (2012 survey 50%, 2015 survey 49%) and for interviews with matching vaccination card 31% (2012 survey 28%, 2015 survey 34%). These proportions are calculated as follows: the proportion of observed eligible subjects was 2132 out of 3054 (70%) in 2012 and 2294 out of 2827 (81%) in 2015; these proportions were multiplied by the number of subjects with whom no contact could be established (*N* = 886 in 2012 and *N* = 934 in 2015) or with whom no communication was possible (*N* = 155 in 2012 and *N* = 37 in 2015), e.g. 1041 in 2012 and 971 in 2015; these estimates were added to the number of observed eligible persons, leading to the estimated number of eligible subjects: (1041 times 2132/3054) plus 2132 in 2012 and (971 times 2294/2827) plus 2294 in 2015, i.e. 100% of the response rate (Fig. 1). The complete combined dataset includes 2942 observations (2011 survey *N* = 1421, 2015 survey *N* = 1521). We included 1851 observations with valid vaccination documents in the analysis (2012 survey: *N* = 803, 43%; 2015 survey: *N* = 1048, 57%).The two survey populations were slightly different with respect to birth year, age, sex and having children (Table 1).

**Table 1:** Demographic characteristics, measles surveys 2012 and 2015, Switzerland

	Survey 2012		Survey 2015		Total		*p* value
	*N*	%	*N*	%	*N*	%	
Sex							
Female	448	56	528	50	976	53	
Male	355	44	520	50	875	47	
Total	803	100	1048	100	1851	100	< 0.05
Age group							
< 25 years	403	50	687	66	1090	59	
> 24 years	400	50	361	34	761	41	
Total	803	100	1048	100	1851	100	< 0.05
Birth cohort							
≥ 1990	226	28	768	73	994	54	
< 1990	576	72	280	27	856	46	
Missing	1	0	0	0	1	0	
Total	803	100	1048	100	1851	100	< 0.05
Nationality							
Swiss	751	94	982	94	1733	94	
Foreign	52	6	66	6	118	6	
Total	803	100	1048	100	1851	100	0.87
Language region							
German	430	54	557	53	987	53	
French	373	46	491	47	864	47	
Total	803	100	1048	100	1851	100	0.86
Educational level							
Secondary	565	70	776	74	1341	72	
Tertiary	236	29	271	26	507	27	
Missing	2	0	1	0	3	0	
Total	803	100	1048	100	1851	100	0.08
Having own children							
Yes	125	16	64	6	189	10	
No	678	84	984	94	1662	90	
Total	803	100	1048	100	1851	100	< 0.05

We validated the question about vaccination by comparing the information given by memory and written on the vaccination card. The agreement was poor (see Table 2).

**Table 2:** Agreement between anamnesis and documented measles vaccination by use of the vaccination document at interview, measles survey 2012 and 2015, Switzerland

	Documented number of doses				
	0	1	2	3	Total
Vaccination document used during interview					
Have you been vaccinated against measles?					
Yes	5 (1.2%)	48 (11.8%)	304 (74.5%)	34 (8.3%)	391 (95.8%)
No	5 (1.2%)	2 (0.5%)	6 (1.5%)	1 (0.2%)	14 (3.4%)
I do not know	1 (0.2%)	0 (0%)	2 (0.5%)	0 (0%)	3 (0.7%)
Total					408 (100%)
Vaccination document not used during interview					
Have you been vaccinated against measles?					
Yes	26 (1.8%)	122 (8.4%)	886 (61.3%)	91 (6.3%)	1125 (77.8%)
No	34 (2.4%)	19 (1.3%)	56 (3.9%)	2 (0.1%)	111 (7.7%)
I do not know	13 (0.9%)	21 (1.5%)	159 (11.0%)	17 (1.2%)	210 (14.5%)
Total					1446 (100.0%)

### Vaccination coverage

**Fig. 2:**
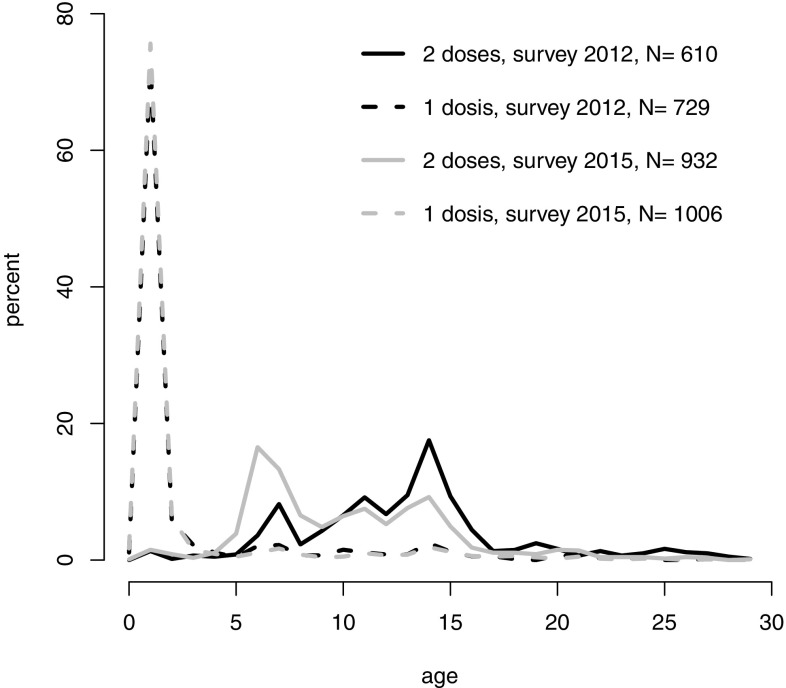
Age distribution at first and second dose of vaccination, 2012 and 2015 measles surveys, Switzerland

Vaccination coverage with at least two doses increased from 77% (95% CI 73–81%) in 2012 to 88% (95% CI 85–90%) in 2015. Only a minority was vaccinated with just one dose and the proportion decreased from 16% (95% CI 13–19%) in 2012 to 9% in 2015 (95% CI 7–12%) in favour of being vaccinated with more than one dose (Table 3). Most participants received their first measles vaccination according to the recommendations: the median age at uptake was 1 year, both in 2012 (interquartile range 1–2 years, *N* = 729) and in 2015 (interquartile range 1–1 year, *N* = 1006). The median age at the second vaccination was 13 years in 2012 (interquartile range 10–15 years, *N* = 610) and 10 years in 2015 (interquartile range 7–13 years, *N* = 923). Very few second doses and even fewer first doses were received between the ages of 20 and 29 years (Fig. 2) in both surveys. According to the 2015 survey, only 4% of respondents (37/932) received their second dose after 2011, the year a national measles vaccination campaign was launched, compared to 8% of respondents (46/610) during an equally long time span after 2008 for the 2012 survey (OR = 0.51, 95% CI 0.32–0.81). However, the catch-up needs for second doses 3 years before the start of the respective survey were lower in 2015 (151/932 = 16%) than in 2012 (228/610 = 37%) (OR = 0.32, 95% CI 0.25–0.41). Accordingly, these catch-up vaccinations with a second dose during the campaign covered 25% of the needs in 2015 respondents compared to 20% in 2012 (OR = 1.28, 95% CI 0.76–2.16). There is a strong cohort effect on the complete vaccination coverage (supplementary material Tables 3 and 4).

**Table 3:** Vaccination coverage and knowledge about measles, measles surveys 2012 and 2015, Switzerland

	Survey 2012				Survey 2015			
	*N* ^a^	%	95% CI		*N*	%	95% CI	
Documented measles vaccination								
Not vaccinated	51	7	5	9	30	3	2	4
1 dose	130	16	13	19	82	9	7	12
2 doses	577	71	67	75	836	78	75	81
3 doses	45	6	4	8	100	10	7	12
Vaccinated with at least two doses								
No	181	23	19	27	112	12	10	15
Yes	622	77	73	81	936	88	85	90
Prior measles disease								
Surely yes	55	7	5	10	82	8	6	10
Rather yes	31	5	3	7	32	3	2	5
Rather no	113	12	9	15	176	19	16	23
Surely no	570	70	65	74	723	65	61	69
I do not know	34	6	3	8	35	4	2	6
Can adults get measles?								
Surely yes	280	32	28	36	374	41	37	45
Rather yes	402	53	48	57	529	48	44	52
Rather no	56	7	5	9	72	6	4	8
Surely no	8	0	0	1	11	1	0	1
I do not know	57	8	5	10	62	5	3	6
Can adults catch up missing vaccinations?								
Surely yes	147	18	14	21	200	23	19	26
Rather yes	408	51	47	56	566	51	47	55
Rather no	100	14	11	17	132	14	11	17
Surely no	28	3	1	5	17	1	0	2
I do not know	120	14	11	17	133	11	9	13
I can endanger others if I am not vaccinated								
Complete agreement	345	42	38	47	451	44	41	48
Slight agreement	287	36	31	40	405	37	34	41
Slight disagreement	94	13	10	16	148	15	12	18
Complete disagreement	46	5	3	7	26	2	1	3
I do not know	31	4	2	6	18	1	0	2
Is vaccination against measles mandatory in Switzerland?								
Surely yes	41	5	3	7	60	5	3	6
Yes, I believe so	106	13	10	16	159	13	11	16
No, I do not believe so	281	32	28	36	410	37	34	41
Surely no	317	44	39	48	379	42	38	46
I do not know	58	6	4	7	40	3	2	4
Did you know about the collaboration of Switzerland with the WHO in eliminating measles?								
Yes	287	38	33	42	438	44	40	48
No	516	62	58	67	610	56	52	60
Is measles elimination necessary in Switzerland?								
Surely yes	332	38	34	43	361	34	30	38
Rather yes	285	35	31	39	393	37	33	41
Rather no	102	15	12	18	177	18	15	21
Surely no	40	5	3	7	44	5	3	6
I do not know	44	7	4	9	73	7	5	9
Do you have professional contact to children, pregnant women or sick persons?								
Yes	259	28	24	32	302	25	22	29
No	544	72	68	76	745	75	71	78
Missing	0				1			

Percent taking into account the weighting. Deviations from the total of 100 per cent is due to rounding errors

^a^*N* total: 803 for measles survey 2012, 1048 for measles survey 2015

**Table 4:** Logistic regression models of documented vaccination coverage with at least two doses, weighted and assuming missing completely at random, measles survey 2012 and 2015, Switzerland

	Univariate analysis			Multivariate analysis		
	OR	95% CI		Adj. OR	95% CI	
Survey						
2012	1			1		
2015	2.15	1.54	3.01	1.67	1.15	2.42
Birth cohort						
≥ 1990	1			1		
< 1990	0.32	0.23	0.45	0.41	0.28	0.61
Sex						
Female	1			1		
Male	0.59	0.42	0.81	0.58	0.42	0.82
Nationality						
Swiss	1			n.i.		
Foreign	1.15	0.59	2.23	n.i.		
Language region						
German	1			n.i.		
French	1.11	0.83	1.48	n.i.		
Educational level						
Secondary	1			1		
Tertiary	0.60	0.43	0.86	0.77	0.53	1.12
Having own children						
Yes	1			n.i.		
No	1.48	0.96	2.31	n.i.		
Prior measles						
Surely yes	1			1		
Yes, I believe so	1.43	0.62	3.28	1.69	0.71	4.05
No, I do not believe so	1.79	0.97	3.31	1.83	0.94	3.54
Surely not	2.33	1.40	3.89	2.46	1.43	4.23
I do not know	1.19	0.49	2.91	1.51	0.61	3.77
Can adults get measles?						
Surely yes	1			n.i.		
Rather yes	1.13	0.79	1.62	n.i.		
Rather no	0.98	0.52	1.86	n.i.		
Surely no	0.85	0.25	2.89	n.i.		
I do not know	0.93	0.50	1.74	n.i.		
Can adults catch up missing vaccinations?						
Surely yes	1			n.i.		
Rather yes	1.00	0.66	1.53	n.i.		
Rather no	0.77	0.44	1.36	n.i.		
Surely no	1.65	0.63	4.37	n.i.		
I do not know	0.89	0.51	1.55	n.i.		
I can endanger others if I am not vaccinated						
Complete agreement	1			n.i.		
Slight agreement	0.76	0.52	1.10	n.i.		
Slight disagreement	0.78	0.48	1.27	n.i.		
Complete disagreement	0.74	0.38	1.41	n.i.		
I do not know	1.60	0.40	6.45	n.i.		
Is vaccination against measles mandatory in Switzerland?						
Surely yes	1			n.i.		
Yes, I believe so	2.06	0.73	5.83	n.i.		
No, I do not believe so	0.96	0.38	2.45	n.i.		
Surely no	0.90	0.35	2.27	n.i.		
I do not know	1.33	0.43	4.09	n.i.		
Did you know about the collaboration of Switzerland with the WHO in eliminating measles?						
Yes	1			n.i.		
No	0.99	0.71	1.38	n.i.		
Is measles elimination necessary in Switzerland?						
Surely yes	1			n.i.		
Rather yes	1.06	0.72	1.57	n.i.		
Rather no	0.73	0.46	1.17	n.i.		
Surely no	1.43	0.67	3.04	n.i.		
I do not know	1.03	0.55	1.91	n.i.		
Do you have professional contact to children, pregnant women or sick persons?						
Yes	1			n.i.		
No	0.95	0.65	1.38	n.i.		

*OR* odds ratio, *CI* confidence interval, *n.i.* not included into the multivariate model

### Knowledge about vaccination

Awareness of measles and measles vaccination improved between 2012 and 2015 (Table 3). First, significantly more participants knew that adults can get measles: 32% in 2012 (95% CI 28–36%) were sure that adults can get measles compared to 41% in 2015 (95% CI 37–45%). Second, there was a trend, although not statistically significant, for more participants to be sure that they could get missing vaccinations as adults: from 18% in 2012 (95% CI 14–21%) to 23% in 2015 (95% CI 19–26%). There was practically no change with respect to the other questions on awareness. In both surveys, a similarly low proportion had previously contracted measles. About a quarter of participants had professional contacts to risk groups (Table 3).

### Determinants of vaccination

In the weighted univariate analysis, we identified the following factors as significantly associated with complete vaccination:‘survey’, birth cohort, sex, educational level and prior measles (Table 4). All these factors except educational level remained statistically significant in the multivariate analysis. Thus, the major determinants of complete measles vaccination were the year of the survey, i.e. respondents surveyed in 2015 were more likely to be fully vaccinated than those surveyed in 2012; birth cohort, i.e. persons born in 1990 or after were more likely to be fully vaccinated than those born before 1990; sex, i.e. females were more likely to be fully vaccinated than males; and prior measles, i.e. those who were sure that they had not had measles were more likely to be fully vaccinated than those with measles in their medical history (Table 4).

### Sensitivity analyses

The sensitivity analysis either neglecting the weights, assuming ‘missing completely at random’ or ‘missing at random’ and their combination confirmed the results of the weighted univariate and multivariate analysis (supplementary material Tables 1 and 2).

There is a strong cohort effect on the primary endpoint complete vaccination coverage. If the analysis is restricted to the birth cohorts 1986–1992, the odds ratio between 2012 and 2015 is 1.40 (95% CI 0.87–2.10). In the multivariate analysis, this estimate drops to 1.29 (95% CI 0.82–2.03) (supplementary material Tables 3 and 4).

The participants with and without vaccination cards are different with respect to the factors ‘survey’, age, language region and anamnestic vaccination status (supplementary material Table 5). There were more missing vaccination cards in 2012 compared to 2015. In both surveys, 82% of respondents reported during the telephone interview to be completely vaccinated, which contrasted with information gained from the vaccination cards.

## Discussion

### Main results

While routine monitoring of vaccination coverage is almost universally established for measles in children and adolescents, it generally does not include adults. The results of this study provide the first reliable estimates of measles vaccination coverage in young adults in Switzerland.

We assessed vaccination coverage and determinants of vaccination among young adults by comparing the results of two cross-sectional surveys conducted in 2012 and 2015. Documented complete vaccination increased from 77 to 88%. However, there is no evidence for an association between vaccination and the recent national awareness campaign or increased awareness. Knowing that measles can also affect adults was the only factor that increased between the two surveys, but this knowledge was not related to the likelihood of vaccination.

### Strengths and limitations

The careful sampling mechanism stratified by sex and language region, the adequately high sample size of valid vaccination card-based data, the thorough statistical analysis taking into account various sensitivity analyses for missing data and weighting, as well as application of the identical questionnaire tools make us confident of the quality of the estimate as well as the significant increase in vaccination coverage between 2012 and 2015. However, the marked increase in vaccination coverage is very likely due to a cohort effect. This cohort effect, unexpected at the design stage of the studies, is so strong, that all multivariate analyses have to be interpreted with care. The observed cohort effect is confirmed by the comparison with the values from the external reference the SNVCS, which also shows the increasing values over the study periods of the SNVCS.

The high number of missing vaccination cards—an exclusion criterion of our study—could lead to a selection bias. The contrast between self-reported vaccination status and documented vaccination history confirms our study design, which focused on the collection of vaccination cards and restricted the analysis on these written records. The return rate of vaccination cards (63%) was in the range of the expectation during the design stage and was anticipated in the sample size calculation for interviews with matching vaccination cards. However, there is evidence that subjects with missing vaccination cards are different between the two surveys, with respect to age, language region, and history of measles vaccination. The process of differential missing data between 2012 and 2015 may lead to an overestimation of the marked increase in vaccination coverage.

There are some critical points with respect to our analysis. The participants were asked about vaccinations that often occurred during their early infancy. Anamnestic information about their vaccination status, provided by participants not consulting their vaccination card, was often uncertain (see Table 2). We therefore restricted our analyses to individuals who provided a vaccination card, thereby reducing response rate from 50 to 31%. This could have introduced selection bias and led us to overestimate the effect, because we assume that people without a vaccination card are less likely to be vaccinated. However, this was partially addressed by a complex weighting procedure, a common method of dealing with the unit non-response. After weighting, the study population had a similar distribution to the Swiss general population in this age range in regard to age, sex and cantonal distribution.

Our results are in line with the vaccination coverage in the 16-year-old in the SNVCS (Bundesamt für Gesundheit 2016b). They are as well comparable to findings from Germany and France. In a study, conducted 2012 in Germany, vaccination coverage for two doses was found to be 56% in adults aged 20–34 years (Schuster et al. 2015). 80% of these participants relied on their vaccination card to answer. Furthermore, the fact that in our study younger age groups and females were more likely to be vaccinated is consistent with the finding of Schuster et al. (2015). In France, measles vaccination coverage (based on vaccination cards of first-year health care students) was 78% in 2011 (Faure et al. 2013). In another French study in 2015, coverage was 93% in 16–18-year-olds (Buscail and Gagnière 2016).

Our results show that the sharp increase of vaccination coverage in just 3 years was very likely due to a “cohort effect” resulting from a change in recommendations made in 1996: a second dose of measles vaccine was introduced to the national vaccination plan for 4–7-year-old children, together with the recommendation to catch up at any age. Only four out of ten birth cohorts (1989–1992) among the participants in the first survey had the opportunity to receive a second dose as routine vaccination, compared to seven cohorts (1989–1995) of participants in the second survey in 2015. In addition, it took a few years for the second dose to become a well-established practice. The gap of catch-up vaccinations with a second dose 3 years before the surveys was therefore larger in the group surveyed in 2012 than that in 2015.

Most of these catch-up vaccinations were administered before the age of 16 years. They were received before the recent promotion campaign and should be credited to paediatricians, school health services and parents. Although one quarter of the pre-campaign vaccination gaps were filled during the campaign, this proportion was not significantly higher than during the 3 years before the campaign suggesting that the campaign had little to no effect on the number of catch-up doses administered.

We estimated the number of catch-up vaccinations administered by primary care physicians in a study within the Swiss Sentinella network (Bundesamt für Gesundheit 2016c). In 2014 and 2015, estimated totals of 33,000 and 37,000 doses of measles catch-up vaccinations were administered by general practitioners and paediatricians. This corresponds to 37% of all doses needed in 2015 to close the estimated gap (with a 95% objective) in measles vaccination for 2–51-year-olds in Switzerland. Two-thirds of these catch-up vaccinations were given to adults aged 20 years and older. Eighty-eight percent of all vaccinations were initiated by the physicians, underlining their crucial role in promoting vaccination and filling the gaps.

Awareness of measles has increased slightly in our study and can almost be considered as “common sense”, with 89% of all people knowing that measles can also affect adults. There mere fact that the study topic was measles elimination and the interviewees were primed by an invitation letter sent out by the FOPH may lead to overestimating results about knowledge and attitude towards measles elimination. Knowledge and attitude questions were highly correlated in our survey. But the data were too scarce to be stratified by all variables and evaluated in an integrated way. In addition, there is no simple and direct causal pathway between knowledge, attitude and actual vaccination; this leads to an inconclusive effect, a problem that was encountered by Larson et al. (2014).

### Conclusion

An 11% increase in measles vaccination coverage within a 3-year period in a population where vaccination coverage is already high is a historic finding. This marked increase in measles vaccination coverage is, however, due to a cohort effect, owing to the introduction of the second dose of vaccine in 1996 in the national vaccination plan. This study provides evidence of an improvement in the awareness about measles and measles vaccination in young adults, which may result in an impact on measles vaccination coverage in the future. The achievement of this marked increase in measles vaccination coverage merits acknowledgment of the efforts made by general practitioners and paediatricians in Switzerland, who remain the main and the most important promoters for measles vaccination. Governments at all levels are challenged to use their leeway to support any efforts made by Swiss physicians to promote vaccination in general.

## Electronic supplementary material

Below is the link to the electronic supplementary material.

Supplementary material 1 (PDF 38 kb)
