# Supplementary material for: Marked increase in measles vaccination coverage among young adults in Switzerland: a campaign or cohort effect?
Source: Int J Public Health. 2018 Apr 19;63(5):589–99. doi: 10.1007/s00038-018-1102-x (PMC5976707; doi:10.1007/s00038-018-1102-x)
Supplement: Supplementary file 1 — Supplementary material 1 (PDF 38 kb) [file 38_2018_1102_MOESM1_ESM.pdf]

Electronic supplementary material:

International Journal of Public Health

Marked increase in measles vaccination coverage among young adults in Switzerland - a campaign or cohort effect?

Altpeter Ekkehardt[1], Wymann Monica[1], Richard Jean-Luc[1], Mäusezahl-Feuz Mirjam[1]

[1] Federal Office of Public Health, Division of Communicable Diseases, Berne, Switzerland

Corresponding author: Ekkehardt Altpeter, ekkehardt.altpeter@bag.admin.ch

Supplementary Table 1: Sensitivity analysis, multivariate logistic regression of measles vaccination with at least 2 documented doses, measles survey 2012 and 2015, Switzerland												
Model 1	unweighted						weighted					
	MAR			MCAR			MAR			MCAR		
	adj. OR	95% CI		adj. OR	95% CI		adj. OR	95% CI		adj. OR	95% CI	
Survey												
2012	1			1			1			1		
2015	1.57	1.19	2.08	1.72	1.28	2.30	1.50	1.05	2.14	1.67	1.15	2.42
Birth cohort												
>=1990	1			1			1			1		
<1990	0.43	0.31	0.59	0.41	0.30	0.56	0.41	0.28	0.61	0.41	0.28	0.61
Sex												
female	1			1			1			1		
male	0.64	0.51	0.80	0.70	0.54	0.91	0.55	0.41	0.75	0.58	0.42	0.82
Educational level												
secondary	1			1			1			1		
tertiary	0.92	0.72	1.17	0.89	0.66	1.19	0.87	0.60	1.25	0.77	0.53	1.12
Prior measles												
surely yes	1			1			1			1		
yes, I believe so	1.34	0.76	2.34	1.26	0.63	2.57	1.59	0.84	3.03	1.69	0.71	4.05
no, I don't believe so	2.05	0.99	4.21	1.98	1.19	3.28	2.02	0.72	5.65	1.83	0.94	3.54
surely not	2.65	1.81	3.88	2.72	1.76	4.14	2.55	1.42	4.58	2.46	1.43	4.23
I don't know	1.63	0.87	3.05	1.58	0.79	3.25	1.56	0.70	3.49	1.51	0.61	3.77
Model 2												
Survey												
2012	1			1			1			1		11
2015	1.57	1.18	2.08	1.71	1.27	2.29	1.51	1.06	2.14	1.69	1.16	2.46
Birth cohort												
>=1990	1			1			1			1		
<1990	0.43	0.31	0.58	0.40	0.29	0.55	0.41	0.28	0.61	0.41	0.27	0.61
Sex												
female	1			1			1			1		
male	0.64	0.51	0.81	0.72	0.55	0.93	0.55	0.40	0.75	0.59	0.42	0.84
Educational level												
secondary	1			1			1			1		
tertiary	0.89	0.69	1.14	0.85	0.63	1.14	0.85	0.58	1.23	0.75	0.51	1.10
Prior measles												
surely yes	1			1			1			1		
yes, I believe so	1.33	0.76	2.34	1.32	0.66	2.72	1.56	0.82	2.99	1.70	0.71	4.08
no, I don't believe so	2.09	1.03	4.23	2.01	1.21	3.34	2.05	0.75	5.66	1.83	0.93	3.58
surely not	2.63	1.79	3.86	2.67	1.73	4.09	2.51	1.40	4.52	2.38	1.39	4.08
I don't know	1.64	0.87	3.08	1.63	0.81	3.38	1.59	0.72	3.53	1.59	0.63	4.02
I can endanger others, if I am not vaccinated												
complete agreement	1			1			1			1		
slight agreement	0.79	0.54	1.17	0.76	0.57	1.03	0.81	0.50	1.32	0.75	0.51	1.12
slight disagreement	0.76	0.50	1.15	0.66	0.44	1.00	0.83	0.48	1.42	0.79	0.46	1.34
complete disagreement	0.58	0.32	1.03	0.47	0.27	0.87	0.71	0.32	1.59	0.74	0.37	1.46
I don't know	1.26	0.43	3.74	2.09	0.81	7.16	1.27	0.38	4.28	1.84	0.42	8.09
Model 3												
Survey												
2012	1			1			1			1		
2015	2.22	1.77	2.78	2.51	1.94	3.28	2.00	1.49	2.70	2.3	1.63	3.23
sex												
female	1			1			1			1		
male	0.66	0.53	0.82	0.74	0.57	0.96	0.55	0.41	0.75	0.61	0.43	0.85
educational level												
secondary	1			1			1			1		
tertiary	0.71	0.56	0.90	0.65	0.49	0.86	0.68	0.47	0.98	0.58	0.40	0.83
prior measles												
surely yes	1			1			1			1		
yes, I belief so	1.32	0.76	2.31	1.22	0.62	2.48	1.59	0.84	3.01	1.64	0.69	3.92
no, I don't believ so	2.11	1.06	4.19	1.96	1.18	3.23	2.06	0.77	5.50	1.77	0.94	3.35
surely not	2.79	1.91	4.08	2.72	1.77	4.13	2.71	1.54	4.76	2.45	1.47	4.09
I don't know	1.62	0.87	3.01	1.56	0.79	3.21	1.59	0.73	3.46	1.54	0.63	3.77
I can endanger others, if I am not vaccinated												
complete agreement	1			1			1			1		
slight agreement	0.81	0.55	1.19	0.78	0.58	1.04	0.82	0.51	1.33	0.76	0.51	1.13
slight disagreement	0.79	0.52	1.19	0.70	0.47	1.05	0.85	0.50	1.45	0.81	0.48	1.35
complete disagreement	0.57	0.32	1.02	0.49	0.28	0.90	0.69	0.31	1.54	0.76	0.39	1.50
I don't know	1.25	0.43	3.65	2.09	0.81	7.13	1.31	0.39	4.44	1.92	0.43	8.59
Model 4												
birth cohort												
>=1990	1			1			1			1		
<1990	0.35	0.27	0.45	0.31	0.23	0.41	0.35	0.25	0.49	0.33	0.23	0.47
sex												
female	1			1			1			1		
male	0.65	0.52	0.82	0.72	0.56	0.94	0.55	0.40	0.75	0.59	0.42	0.83
educational level												
secondary	1			1			1			1		
tertiary	0.93	0.73	1.18	0.90	0.68	1.21	0.89	0.62	1.28	0.80	0.55	1.17
prior measles												
surely yes	1			1			1			1		
yes, I belief so	1.29	0.73	2.26	1.30	0.65	2.66	1.49	0.78	2.83	1.58	0.66	3.77
no, I don't believ so	2.10	1.04	4.22	2.03	1.22	3.36	2.10	0.77	5.72	1.91	0.97	3.77
surely not	2.56	1.74	3.77	2.62	1.69	3.98	2.43	1.34	4.41	2.32	1.34	3.99
I don't know	1.62	0.86	3.03	1.59	0.79	3.28	1.53	0.68	3.43	1.50	0.59	3.85
I can endanger others, if I am not vaccinated												
complete agreement	1			1			1			1		
slight agreement	0.80	0.54	1.17	0.76	0.57	1.02	0.81	0.50	1.32	0.75	0.51	1.11
slight disagreement	0.76	0.50	1.16	0.67	0.45	1.01	0.82	0.48	1.42	0.79	0.47	1.35
complete disagreement	0.56	0.31	0.99	0.44	0.25	0.80	0.69	0.31	1.54	0.68	0.35	1.33
I don't know	1.24	0.42	3.67	1.96	0.75	6.70	1.18	0.34	4.09	1.60	0.37	6.97

MAR: missing at random
MCAR: missing completly at random
adj. OR: adjusted odds ratio
95%CI: 95% confidence interval

Supplementary Table 2: Sensitivity analysis, univariate logistic regression of measles vaccination with at least 2 documented doses, measles survey 2012 and 2015, Switzerland

	unweighted						weighted					
	MAR			MCAR			MAR			MCAR		
	OR	95% CI		OR	95% CI		OR	95% CI		OR	95% CI	
Survey												
2012	1			1			1			1		
2015	2.14	1.72	2.67	2.43	1.88	3.15	1.91	1.43	2.56	2.15	1.54	3.01
Birth cohort												
>=1990	1			1			1			1		
<1990	0.35	0.28	0.45	0.32	0.24	0.42	0.34	0.24	0.48	0.32	0.23	0.45
Sex												
female	1			1			1			1		
male	0.69	0.56	0.85	0.77	0.60	0.98	0.55	0.41	0.74	0.59	0.43	0.81
Nationality												
Swiss	1			1			1			1		
foreign	0.89	0.51	1.54	0.92	0.57	1.55	0.96	0.40	2.32	1.15	0.59	2.23
Language region												
German	1			1			1			1		
French	1.19	0.97	1.46	1.05	0.82	1.34	1.23	0.98	1.55	1.11	0.83	1.48
Educational level												
secondary	1			1			1			1		
tertiary	0.77	0.61	0.97	0.68	0.53	0.90	0.75	0.53	1.08	0.60	0.43	0.86
Having own children												
yes	1			1			1			1		
no	1.7	1.3	2.3	2.2	1.5	3.1	1.4	0.9	2.0	1.5	1.0	2.3
Prior measles												
surely yes	1			1			1			1		
yes, I believe so	1.1	0.7	1.9	1.0	0.5	2.1	1.4	0.7	2.5	1.4	0.6	3.3
no, I don't believe so	2.0	1.0	3.9	1.8	1.1	3.0	2.0	0.8	5.3	1.8	1.0	3.3
surely not	2.6	1.8	3.7	2.5	1.7	3.7	2.6	1.5	4.6	2.3	1.4	3.9
I don't know	1.4	0.8	2.6	1.3	0.7	2.5	1.4	0.6	3.1	1.2	0.5	2.9
Can adults get measles?												
surely yes	1			1			1			1		
rather yes	1.30	1.02	1.66	1.41	1.07	1.85	1.11	0.75	1.63	1.13	0.79	1.62
rather no	0.92	0.62	1.38	1.12	0.69	1.91	0.84	0.48	1.46	0.98	0.52	1.86
surely no	0.97	0.25	3.73	0.83	0.29	2.94	1.00	0.12	8.73	0.85	0.25	2.89
I don't know	0.77	0.51	1.18	0.87	0.54	1.45	0.80	0.45	1.40	0.94	0.50	1.74
Can adults catch up missing vaccinations?												
surely yes	1			1			1			1		
rather yes	1.10	0.79	1.53	1.10	0.78	1.53	1.06	0.73	1.53	1.00	0.66	1.53
rather no	0.98	0.65	1.48	0.98	0.63	1.55	0.81	0.47	1.40	0.77	0.44	1.36
surely no	0.76	0.24	2.44	0.89	0.41	2.15	0.84	0.19	3.68	1.65	0.63	4.37
I don't know	0.82	0.58	1.17	0.87	0.57	1.33	0.81	0.51	1.26	0.89	0.51	1.55
I can endanger others, if I am not vaccinated												
complete agreement	1			1			1			1		
slight agreement	0.84	0.56	1.23	0.78	0.59	1.04	0.85	0.53	1.38	0.76	0.52	1.10
slight disagreement	0.78	0.52	1.17	0.71	0.49	1.06	0.81	0.49	1.35	0.78	0.48	1.27
complete disagreement	0.55	0.31	0.95	0.44	0.26	0.79	0.67	0.31	1.44	0.74	0.38	1.41
I don't know	1.14	0.41	3.15	1.78	0.71	6.01	1.17	0.36	3.78	1.60	0.40	6.45
Is vaccination against measles mandatory in Switzerland?												
surely yes	1			1			1			1		
yes, I believe so	1.53	0.70	3.35	1.35	0.63	2.77	2.23	0.98	5.06	2.06	0.73	5.83
no, I do not believe so	0.78	0.45	1.37	0.71	0.36	1.30	1.06	0.51	2.20	0.96	0.38	2.45
surely no	0.65	0.40	1.05	0.58	0.30	1.05	0.96	0.47	1.94	0.90	0.36	2.27
I don't know	0.81	0.41	1.62	0.69	0.30	1.54	1.36	0.37	5.08	1.33	0.43	4.09
Did you know about the collaboration of Switzerland with the WHO in eliminating measles?												
yes	1			1			1			1		
no	1.19	0.91	1.55	1.17	0.91	1.50	1.03	0.71	1.49	0.99	0.71	1.38
Is measles elimination necessary in Switzerland?												
surely yes	1			1			1			1		
rather yes	1.14	0.79	1.64	1.20	0.89	1.62	1.08	0.67	1.74	1.06	0.72	1.57
rather no	0.76	0.56	1.02	0.80	0.56	1.14	0.70	0.46	1.07	0.74	0.46	1.17
surely no	0.88	0.53	1.46	0.95	0.53	1.82	1.04	0.57	1.90	1.43	0.67	3.04
I don't know	0.75	0.46	1.20	0.87	0.53	1.49	0.79	0.44	1.42	1.03	0.55	1.91
Do you have professional contact to children, pregnant women or sick persons?												
yes	1			1			1			1		
no	0.98	0.73	1.33	0.97	0.73	1.26	0.91	0.60	1.36	0.95	0.65	1.38

Supplementary Table 3: Vaccination coverage with 2 doses by birth cohort 1982 to 1995 at 16-years and 20- to 29-years weighted and assuming missing completly at random, measles survey 2012 and 2015, Switzerland

birth cohort	survey 2012	survey 2015	Total	vaccinated with 2 doses at time of survey 2012	standard error	vaccinated with 2 doses until age of 16-years, survey 2012	standard error	vaccinated with 2 doses at time of survey 2015	standard error	vaccinated with 2 doses until age of 16-years, survey 2015	standard error	vaccinated with 2 doses at time of survey, pooled	standard error	vaccinated with 2 doses until age of 16-years, pooled	standard error	SNCVS* vaccinated with 2 doses at 16-years	catchup, pooled**	standard error
1982	58	0	58	55%	(±8%)	42%	(±8%)	NA	NA	NA	NA	55%	(±8%)	42%	(±8%)	NA	13%	(±6%)
1983	95	0	95	61%	(±6%)	55%	(±6%)	NA	NA	NA	NA	61%	(±6%)	55%	(±6%)	54%	6%	(±2%)
1984	76	0	76	70%	(±7%)	59%	(±7%)	NA	NA	NA	NA	70%	(±7%)	59%	(±7%)	54%	11%	(±4%)
1985	77	0	77	76%	(±6%)	73%	(±6%)	NA	NA	NA	NA	76%	(±6%)	73%	(±6%)	54%	3%	(±2%)
1986	70	64	134	71%	(±7%)	65%	(±7%)	76%	(±7%)	61%	(±8%)	73%	(±5%)	63%	(±5%)	54%	10%	(±3%)
1987	52	62	114	71%	(±9%)	62%	(±9%)	77%	(±7%)	69%	(±7%)	74%	(±6%)	66%	(±6%)	54%	9%	(±3%)
1988	72	71	143	84%	(±5%)	76%	(±6%)	90%	(±3%)	82%	(±5%)	87%	(±3%)	79%	(±4%)	NA	8%	(±3%)
1989	76	83	159	84%	(±5%)	75%	(±6%)	83%	(±6%)	65%	(±7%)	84%	(±4%)	69%	(±5%)	76%	14%	(±4%)
1990	93	84	177	87%	(±4%)	80%	(±5%)	95%	(±2%)	79%	(±6%)	91%	(±2%)	79%	(±4%)	76%	12%	(±4%)
1991	110	112	222	91%	(±3%)	85%	(±4%)	86%	(±5%)	75%	(±5%)	88%	(±3%)	79%	(±4%)	76%	9%	(±2%)
1992	24	119	143	NA	NA	NA	NA	95%	(±2%)	81%	(±4%)	94%	(±3%)	83%	(±4%)	85%	11%	(±3%)
1993	0	129	129	NA	NA	NA	NA	91%	(±3%)	81%	(±4%)	91%	(±3%)	81%	(±4%)	85%	10%	(±3%)
1994	0	173	173	NA	NA	NA	NA	90%	(±3%)	85%	(±3%)	90%	(±3%)	85%	(±3%)	85%	6%	(±2%)
1995	0	151	151	NA	NA	NA	NA	92%	(±2%)	90%	(±3%)	92%	(±2%)	90%	(±3%)	89%	2%	(±1%)

* External data source, Swiss National Vaccination Coverage Survey

** catch-up vaccination is the difference between the total number of respondents vaccinated with two doses at time of survey minus those, which had been vaccinated with 2 doses at age of 16-years.

"pooled" means that the data are analyzed disregarding the survey.

Supplementary Table 4: Logistic regression models of documented vaccination coverage, weighted and assuming missing completely at random, measles survey 2012 and 2015, Switzerland

	univariate analysis			multivariate analysis		
	OR	95% CI		adj. OR	95% CI	
Survey						
2012	1			1		
2015	2.20	1.50	3.00	1.29	0.82	2.03
Birth cohort						
1982	1			1		
1983	1.30	0.57	2.90	1.30	0.57	3.00
1984	1.90	0.80	4.60	2.13	0.86	5.29
1985	2.70	1.10	6.60	2.99	1.19	7.49
1986	2.30	1.00	5.00	2.22	0.96	5.09
1987	2.40	1.00	5.50	2.24	0.92	5.44
1988	5.60	2.50	12.00	5.43	2.30	12.80
1989	4.20	1.80	9.70	3.78	1.58	9.06
1990	8.60	3.70	20.00	8.37	3.43	20.40
1991	6.20	2.70	14.00	5.68	2.44	13.20
1992	12.00	4.30	33.00	10.80	3.55	32.90
1993	8.60	3.30	22.00	7.31	2.48	21.60
1994	7.70	3.30	18.00	6.45	2.43	17.10
1995	10.00	4.10	25.00	8.59	3.02	24.50
Prior measles						
surely yes	1			1		
yes, I believe so	1.43	0.62	3.28	1.92	0.82	4.52
no, I don't believe so	1.79	0.97	3.31	1.91	0.98	3.71
surely not	2.33	1.40	3.89	2.72	1.57	4.73
I don't know	1.19	0.49	2.91	1.74	0.70	4.36

OR Odds ratio, CI confidence interval

Supplementary Table 5: Evaluation of the outcome "vaccination card absent yes=1 and no=0", measles survey 2012 and 2015, Switzerland

	2012				2015				Total				adj. OR	95% C.I.	
	not missing		missing		not missing		missing		not missing		missing				
Total	803	100%	618	100%	1048	100%	473	100%	1851	100%	1091	100%	0.61*	0.52	0.71
Sex															
women	448	56%	341	55%	528	50%	224	47%	976	53%	565	52%	1		
men	355	44%	277	45%	520	50%	249	53%	875	47%	526	48%	1.10	0.9	1.2
Age in years													1.10	1	1.1
median	24		25		23		24		23		25				
1. quartile	22		22		21		21		21		22				
2. quartile	27		28		26		27		26		27				
Language region															
German	430	54%	281	45%	557	53%	160	34%	987	53%	441	40%	1		
French	373	46%	337	55%	491	47%	313	66%	864	47%	650	60%	1.70	1.5	2
anamnestic vaccination status															
vaccinated	657	82%	435	70%	858	82%	349	74%	1515	82%	784	72%	1		
not vaccinated	51	6%	61	10%	73	7%	56	12%	124	7%	117	11%	1.80	1.4	2.2
no memory	95	12%	122	20%	117	11%	68	14%	212	11%	190	17%	1.70	1.4	2.2
prior measles															
surely yes	55	7%	48	8%	82	8%	41	9%	137	7%	89	8%	1		
believes yes	31	4%	41	7%	32	3%	23	5%	63	3%	64	6%	1.50	0.95	2.4
believes no	113	14%	86	14%	176	17%	75	16%	289	16%	161	15%	0.87	0.61	1.2
surely no	570	71%	422	68%	723	69%	320	68%	1293	70%	742	68%	0.96	0.7	1.3
don't know	34	4%	21	3%	35	3%	14	3%	69	4%	35	3%	0.66	0.39	1.1

* Reference survey 2012
